# Maternal Treatment with Metformin Persistently Ameliorates High-Fat Diet-Induced Metabolic Symptoms and Modulates Gut Microbiota in Rat Offspring

**DOI:** 10.3390/nu14173612

**Published:** 2022-09-01

**Authors:** Lin Song, Jiaqi Cui, Shuyuan Hu, Rui Wang, Hongbao Li, Bo Sun

**Affiliations:** 1 Department of Physiology and Pathophysiology, School of Basic Medical Sciences, Xi’an Jiaotong University Health Science Center, Xi’an 710061, China; 2 Key Laboratory of Environment and Genes Related to Diseases, Ministry of Education of China, Xi’an Jiaotong University, Xi’an 710061, China; 3 Institute of Neuroscience, Translational Medicine Institute, Xi’an Jiaotong University Health Science Center, Xi’an 710061, China; 4 Department of Health Management Center, The First Affiliated Hospital of Xi’an Jiaotong University, Xi’an 710061, China

**Keywords:** maternal diet, metformin, gut microbiota, bile acids, hypothalamus

## Abstract

A maternal high-fat (HF) diet has long-term deleterious effect on offspring. This study aims to evaluate whether maternal metformin (MT) treatment ameliorates the adverse effects of maternal HF diet on offspring and the role of gut microbiota in it. Pregnant Sprague-Dawley rats were randomly assigned to a HF diet (60% fat) or a standard chow diet (11.8% fat) group, and part of the HF diet group rats were co-treated with MT via drinking water (300 mg/kg/day), resulting in three groups according to maternal diet and MT treatment during gestation and lactation. All offspring were weaned on a chow diet. A maternal HF diet showed a significant deleterious effect on offspring’s metabolic phenotype and induced colonic inflammation and gut-barrier disruption through the reshaped gut microbiota. The daily oral administration of MT to HF-fed dams during gestation and lactation reversed the dysbiosis of gut microbiota in both dams and adult offspring. The hypothalamic TGR5 expression and plasma bile acids composition in adult male offspring was restored by maternal MT treatment, which could regulate hypothalamic appetite-related peptides expression and alleviate inflammation, thereby improving male offspring’s metabolic phenotype. The present study indicates that targeting the gut–brain axis through the mother may be an effective strategy to control the metabolic phenotype of offspring.

## 1. Introduction

The hypothesis of the Developmental Origin of Health and Disease (DOHaD) emphasizes that the environmental conditions in early life, such as maternal nutritional status or exposures to drugs, may shape the metabolic health of the offspring in the long term [1,2]. Maternal obesity or maternal high-fat (HF) diet leads to increased incidence of gestational diabetes mellitus (GDM), preeclampsia, stillbirth and increased risk of developing metabolic syndrome in offspring [3,4]. During gestation and lactation periods, maternal high-calorie diets promote obesity, inflammation, insulin resistance and abnormal lipid metabolism in both mother and their offspring [5,6]. Therefore, managing maternal overnutrition is of great importance. 

Maternal metformin (MT) treatment has been shown to reduce insulin resistance and exert anti-inflammatory effects in offspring of HF-diet-fed mothers [6,7]. In addition to this, it is also beneficial in improving maternal–fetal obstetric outcomes in pregnancies of GDM patients [8]. Although MT has been considered safe during the first gestational trimester and widely prescribed for the treatment of GDM [9,10], the long-term effects of MT exposure during early phases of body development are still controversial [11,12,13]. In human studies, MT treatment for GDM [11,14] or polycystic ovary syndrome (PCOS) pregnancies [15,16] was associated with increased childhood adiposity. Opposite to our view, in rodent studies, maternal MT intervention causes increased adiposity [17,18] and inflammation in white adipose tissue [19] in adult offspring of different models. Maternal MT treatment does not program cardiovascular and metabolic alterations [12] but can induce alterations in reproductive parameters of male rat offspring [13]. In addition to the controversial long-term effects of maternal MT treatment, the underlying molecular mechanisms are not completely known.

A non-genetic yet heritable contributor to the intergenerational effect of maternal environment changes may be the gut microbiota, which is related the development and function of the metabolic, immune and central nervous systems (CNS) [20,21,22]. It is believed that the initial gut ecosystem of the offspring originated from the maternal microbiota [23]. Diet and drugs such as MT can rapidly reshape the gut microbiome in human and non-pregnant mice [24,25]. Thus, the influences of maternal HF diet and MT exposure on offspring’s gut microbiota composition may alter the metabolic phenotype in the offspring. 

Given the emerging data that link the maternal microbiome to fetal brain development [26], it seems possible that maternal diet or drug-exposure-induced alterations in the gut microbiome may affect offspring’s metabolic phenotype via the gut–brain axis. The gut microbiota produces a large number of metabolites, which can regulate host metabolism. Bile acids (BAs) are one of the metabolites first synthesized in the liver and further processed by the gut microbiota into secondary BAs [27]. BAs regulate multiple metabolic pathways through the G protein-coupled membrane receptor 5 (TGR5) and the farnesoid X receptor (FXR) [28], and both BAs and their receptors are found in the CNS [29]. Recent studies support the important roles of hypothalamic TGR5 and FXR in the regulation of energy homeostasis and appetite [30,31]. As the hypothalamus (HYP) is the main central regulator of energy balance and is developing during gestation and lactation periods [32], it is natural to question whether maternal diet or MT treatment affects offspring’s metabolic phenotype through the microbiota-BAs-HYP axis. In contrast to humans, hypothalamic neurocircuits development in rodents is not completed at birth but continues until the third week of lactation [33,34]. The number of neuronal cells is determined in utero, whereas the development of axonal projections and synaptic connections occurs during lactation period in rodents [33]. Thus, both gestation and lactation periods are important for hypothalamic development in rodents. In this study, we established a rat model to identify the long-term effect of MT treatment during these critical phases on offspring’s metabolic phenotype in response to maternal HF diet and the role of gut–brain axis in it.

## 2. Materials and Methods

### 2.1. Animals

All animal experiments have been approved by the ethics committee of Xi’an Jiaotong University (No. 2022-1185) and performed in accordance with the national regulations on the administration of experimental animals. Virgin female Sprague-Dawley rats (200–210 g) were received from the Experimental Animal Center of Xi’an Jiaotong University. All rats were housed individually in a room with controlled temperature (22–24 °C) and 12-h light/dark cycle (light onset at 0800) and had free access to standard rat chow and tap water. 

**Figure 1:**
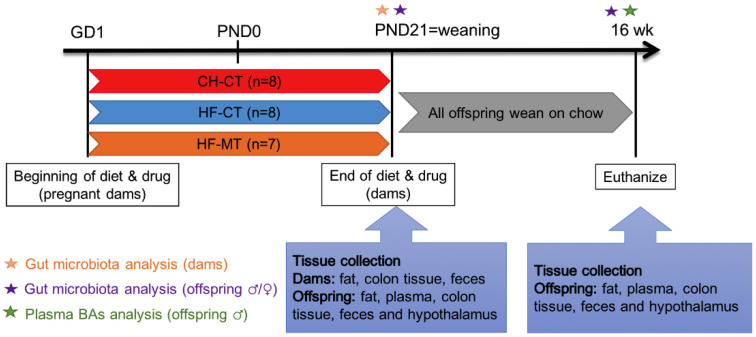
**Schematic of maternal diet and drug regimens and tissue collection time points.** Dams were fed a chow (CH) or high-fat (HF) diet and given water (CT) or metformin (MT) throughout gestation and lactation. Offspring were all weaned on a chow diet and given water without metformin. Tissues, plasma and colonic contents were collected at postnatal day (PND) 21 and 16 weeks of age separately.

All female rats were habituated for one week and then were mated with male rats (280–300 g). Pregnancy was confirmed by the presence of a vaginal plug. The day of vaginal plug expulsion was assigned as gestation day (GD) 1. Pregnant rats were randomized to receive either a control chow diet (CH; Beijing Keao Xieli Feed Co. Ltd., Beijing, China; 11.8% kcal from fat, 65.1% kcal from carbohydrate, 23.1% kcal from protein; *n* = 8) or a high-fat diet (HF; Research Diets D12492, New Brunswick, NJ, USA; 60% kcal from fat, 20% kcal from carbohydrate, 20% kcal from protein; *n* = 15). All dams remained on their respective diets throughout gestation and lactation. The HF-fed dams were then divided into two subgroups with or without MT treatment throughout gestation and lactation. The MT treatment group (HF-MT, *n* = 7) received MT (Sigma-Aldrich, St Louis, MO, USA) administration about 300 mg/kg/d. Normal drinking water was provided to the control groups (CH-CT, *n* = 8 and HF-CT, *n* = 8) (Figure 1). During gestation and lactation, dams’ body weight and food intake were measured weekly. The day of parturition is considered as postnatal day (PND) 0. On PND1, 5 males and 5 females from the same group were culled into one litter. All pups were weighed weekly and weaned on the control CH diet. After weaning, pups were separated by sex, pups from the same group were co-housed (4–5 animals per cage).

### 2.2. Tissue Collection

The animals were fasted for four hours before sacrifice. On PND21, dams, one male and one female per litter were decapitated. At 16 weeks of age, another one male and one female offspring from each litter were sacrificed. The colon content, colon tissue and hypothalamus (bregma −1.56 to −3.48 mm) of both dams and offspring were quickly dissected and immediately frozen in liquid nitrogen and then stored at −80 °C until analysis. Plasma of offspring was collected for target metabolomics analyses. The retroperitoneal (RP) and inguinal subcutaneous (SC) fat were bilaterally dissected and weighed from both dams and offspring at PND21 and 16 weeks old. 

### 2.3. Quantitative Real-Time PCR Analysis

Total RNA was extracted from the colon and hypothalamus samples using RNA isolation kit (R0027, Beyotime, Beijing, China) and then reverse transcribed to cDNA with Reverse Transcription Kit (K1622, Thermo Scientific, Waltham, MA, USA). Gene expression was quantified by qPCR using SYBR Green Pro Taq HS (AG11701, Accurate Biology, Changsha, Hunan, China) in an iQ5 PCR cycler (Bio-Rad, Hercules, CA, USA) with specific primers. All the results were analyzed using the −ΔΔCt method and normalized to the reference gene *Actb*. The primer sequences are listed in Table 1. 

### 2.4. 16S rDNA Sequencing and Data Analysis 

The collected fecal pellets were sent to GENEWIZ, Inc. (Suzhou, China) for 16S rDNA sequencing. In brief, total genome DNA were extracted from the feces. To generate V3-V4 amplicons of the 16S rRNA gene, DNA sequences were subjected with PCR using primers and then sequenced by Illumina MiSeq/NovaSeq platform (Illumina, San Diego, CA, USA). The generated and demultiplexed sequences were analyzed by the QIIME data analysis package. Sequences were clustered into operational taxonomic units (OTUs) using the VSEARCH clustering (1.9.6) program against the Silva 138 database preclustered at 97% sequence identity. Alpha diversity and principal coordinate analysis (PCoA) plots were implemented using QIIME based on the results of OTU analysis. Linear discriminate analysis (LDA) Effect Size (LEfSe) was used to estimate taxonomic abundance and characterize differences among groups. LDA scores (>3.0) derived from LEfSe analysis were used to show the characterized taxon in each group. 

### 2.5. Targeted Metabolomics Analysis 

The plasma was sent to Metware Biological Technology Co., Ltd. (Wuhan, China) for BA detection by lipid chromatograph/mass spectrometry (LC-MS) based on the AB Sciex QTRAP 6500 LC-MS/MS platform. The BA data was normalized using the range method, and R software (3.5.0, www.r-project.org, accessed on 15 September 2021) was used to perform the principal component analysis (PCA) and the hierarchical cluster analysis (HCA) on the accumulation pattern of metabolites between different groups.

### 2.6. Statistical Analysis

All data are presented as the mean ± SEM. Statistical analysis was performed by GraphPad Prism 8.0 (GraphPad, San Diego, CA, USA). Repeated measures ANOVA or one-way ANOVA followed by Tukey’s post hoc comparation was used to compare differences among multiple groups. Results were considered significant when *p* < 0.05. One male and one female offspring was taken from each litter for each experimental set to avoid any litter effects (CH-CT, 8 litters; HF-CT, 8 litters; HF-MT, 7 litters). 

## 3. Results

### 3.1. Maternal MT Treatment Improves HF-Fed Dams’ and Offspring’s Metabolic Phenotype

**Figure 2:**
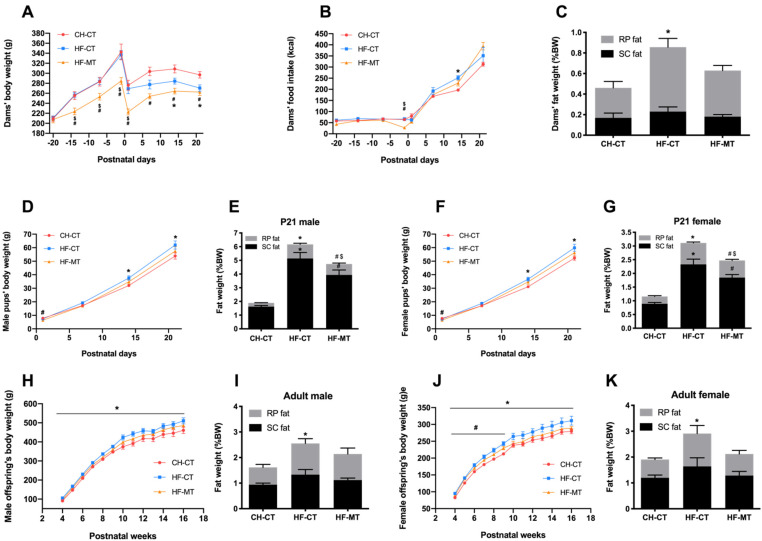
**Maternal MT treatment improves HF**−**fed dams’ and offspring’s metabolic phenotype:** (**A**,**B**) dams’ body weight and food intake during gestation and lactation; (**C**) dams’ subcutaneous (SC) and retroperitoneal (RP) fat weight (% body weight) at postnatal day 21; (**D**,**F**) body weight of male and female offspring during lactation; (**E**,**G**) male and female pups’ SC and RP fat weight (% body weight) at weaning; (**H**,**J**) body weight of male and female offspring from 4 to 16 weeks old; (**I**,**K**) male and female offspring’ SC and RP fat weight (% body weight) at 16 weeks of age. Data are presented as the mean ± SEM. CH-CT, *n* = 8; HF-CT, *n* = 8; HF-MT, *n* = 7. Statistical analyses were performed using repeated ANOVA or one-way ANOVA with Tukey’s post hoc tests. * *p* < 0.05, HF-CT vs. CH-CT; ^#^ *p* < 0.05, HF-MT vs. CH-CT; ^$^ *p* < 0.05, HF-MT vs. HF-CT.

During gestation, HF-fed dams treated with MT had significantly decreased body weight compared with the CH-CT and HF-CT groups (Figure 2A). HF-MT dams still had significantly lower body weight compared with the control group during lactation, while the body weight of HF-CT dams was marked decreased during the last week of lactation when compared with the CH-CT group (Figure 2A). The food intake of HF-MT dams only decreased before giving birth compared with the CH-CT and HF-CT groups, while the HF-fed dams had increased food intake at PND14 compared with the control group (Figure 2B). Though the HF-CT dams had decreased body weight at weaning, the RP fat weight was significantly increased in these dams compared with the CH-CT dams (Figure 2C). However, maternal MT treatment improved the body fat composition in HF-fed dams (Figure 2C).

In both male and female pups, maternal HF diet significantly increased the body weight (Figure 2D,F) and both RP and SC fat weight (Figure 2E,G) at weaning compared with the CH-CT group. The HF-MT offspring had significantly lower body weight at PND1 compared with the CH-CT group (Male, CH-CT: 7.9 ± 0.1 g vs. HF-MT: 6.4 ± 0.3 g; Female, CH-CT: 7.7 ± 0.1 g vs. HF-MT: 6.5 ± 0.4 g; Figure 2D,F). However, the pups’ body weight at PND7, PND14 and PND21 were similar between these two groups (Figure 2D,F). The HF-MT offspring had increased adipose depots compared with the control group (Figure 2E,G), however, the RP fat weight in HF-MT pups was decreased when compared with the HF-CT group at weaning (Figure 2E,G). As expected, maternal HF diet had a long-lasting effect on body weight in both male and female offspring after weaning and through adulthood (Figure 2H,J). The HF-CT offspring also had higher RP fat weight compared with the control group at 16 weeks of age (Figure 2I,K). Interestingly, female HF-MT offspring showed elevated body weight from 4 to 8 weeks of age compared with the CH-CT group (Figure 2J). Maternal MT treatment normalized the body fat composition in male and female offspring from HF-fed dams at 16 weeks of age (Figure 2I,K).

### 3.2. Maternal MT Treatment Restores Gut Integrity and Inflammatory Conditions in Both HF-Fed Dams and Offspring 

**Figure 3:**
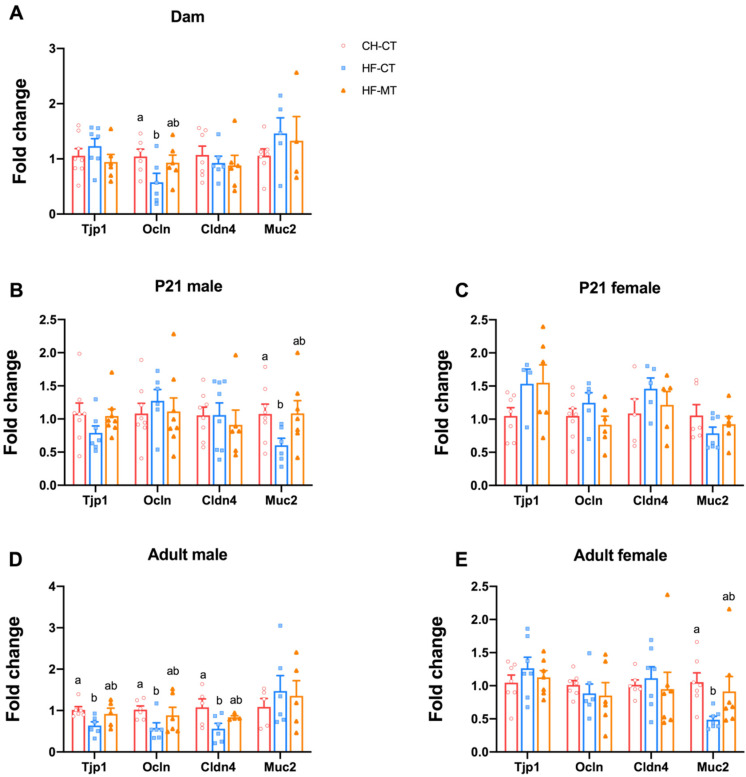
**Maternal metformin treatment restores gut integrity in response to maternal high-fat diet in dams and offspring:** (**A**) colonic mRNA expression of tight junction proteins including the tight junction protein 1 (Tjp1), occlucin (Ocln), claudin 4 (Cldn4) and mucin 2 (Muc2) in dams at weaning; (**B**,**C**) mRNA expression of tight junction proteins in colon of male and female offspring at weaning; (**D**,**E**) mRNA expression of tight junction proteins in colon of male and female offspring at 16 weeks old. Data are presented as the mean ± SEM. CH-CT, *n* = 6–8; HF-CT, *n* = 5–7; HF-MT, *n* = 4–7. Statistical significance was determined using one-way ANOVA with Tukey’s post hoc tests. Groups with different superscript letters differ from each other at *p* < 0.05 level.

To evaluate the integrity of the intestinal epithelial cells, the colonic mRNA expression of tight junction proteins including the tight junction protein 1 (Tjp1, encoded by *Tjp1*), occlucin (Ocln, encoded by *Ocln*), claudin 4 (Cldn4, encoded by *Cldn4*) and mucin 2 (Muc2, encoded by *Muc2*) was investigated. In dams, mRNA expression of Ocln was decreased in the proximal colon of the HF-CT group and was restored by maternal MT treatment at weaning (Figure 3A). The mRNA expression of Muc2 in PND21 male pups and mRNA expression of Tjp1, Ocln and Cldn4 in adult male offspring were significantly decreased in the HF-CT group, while maternal MT treatment normalized them (Figure 3B,D). In female offspring, the gene expression of these tight junction proteins was not altered by maternal HF diet or MT treatment at PND21 (Figure 3C). However, the mRNA expression of Muc2 was downregulated in the HF-CT group and was restored in the HF-MT group in adult females (Figure 3E). 

**Figure 4:**
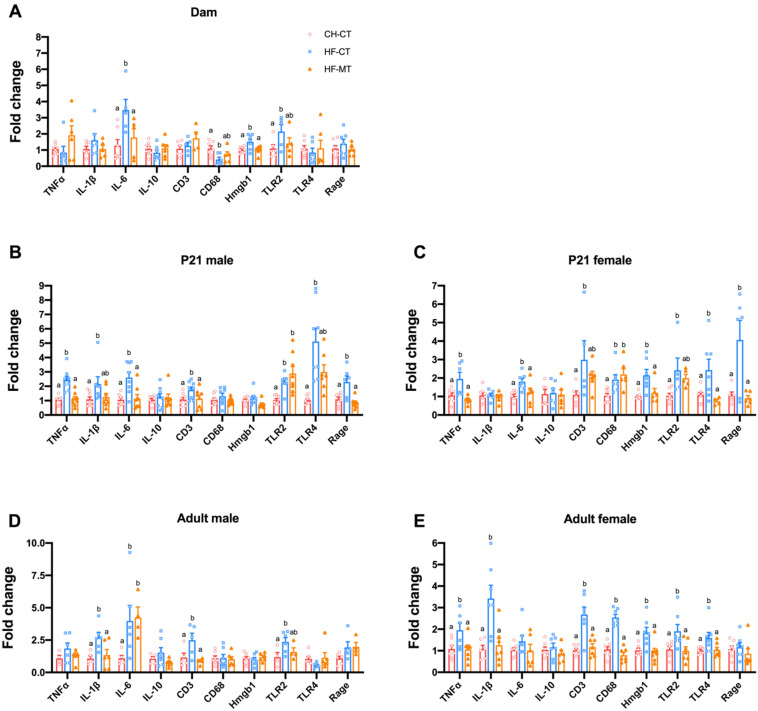
**Maternal metformin treatment attenuates maternal high-fat diet induced gut inflammation in dams and offspring:** (**A**) colonic mRNA expression of inflammatory markers including tumor necrosis factor alpha (TNFα), interleukin 1 beta (IL-1β), interleukin 6 (IL-6), interleukin 10 (IL-10), CD3, CD68, toll-like receptor 2 (TLR2), toll-like receptor 4 (TLR4), high mobility group protein 1 (Hmgb1) and receptor for advanced glycosylation end product-specific receptor (RAGE) in dams at weaning; (**B**,**C**) colonic mRNA expression of inflammatory markers in male and female offspring at weaning; (**D**,**E**) colonic mRNA expression of inflammatory markers in male and female offspring at 16 weeks old. Data are presented as the mean ± SEM. CH-CT, *n* = 5–8; HF-CT, *n* = 5–8; HF-MT, *n* = 4–7. Statistical significance was determined using one-way ANOVA with Tukey’s post hoc tests. Groups with different superscript letters differ from each other at *p* < 0.05 level.

To investigate the gut inflammatory status, we measured colonic mRNA expression of tumor necrosis factor alpha (TNFα, encoded by *Tnfa*), interleukin 1 beta (IL-1β, encoded by *Il1b*), interleukin 6 (IL-6, encoded by *Il6*), interleukin 10 (IL-10, anti-inflammatory cytokine, encoded by *Il10*), CD3 (a marker for T lymphocytes, encoded by *Cd3*), CD68 (a marker for macrophages, encoded by *Cd68*), toll-like receptor 2 (TLR2, a receptor for Gram-positive bacterial lipoteichoic acid, encoded by *Tlr2*), toll-like receptor 4 (TLR4, a receptor for Gram-negative bacterial lipopolysaccharide, encoded by *Tlr4*), high mobility group protein 1 (Hmgb1, an inflammatory effector downstream of TLR2 and TLR4, encoded by *Hmgb1*) and receptor for advanced glycosylation end product-specific receptor (RAGE, encoded by *Ager*). In dams, maternal MT treatment alleviated the increased gene expression of IL-6, Hmgb1 and TLR2 in HF-fed dams at weaning (Figure 4A). In offspring, maternal HF diet increased expression of these inflammatory genes in both male (TNFα, IL-1β, IL-6, CD3, TLR2, TLR4 and Rage) and female (TNFα, IL-6, CD3, CD68, Hmgb1, TLR2, TLR4 and Rage) offspring on PND21, while maternal MT treatment normalized the expression of these genes (except for CD68 in females) (Figure 4B,C). The anti-inflammatory effects of maternal MT treatment continued into adulthood in both male and female offspring (Figure 4D,E). 

In short, these data reveal that maternal MT treatment could restore gut-barrier function and alleviate inflammatory damage induced by maternal HF diet in both dams and their offspring.

### 3.3. Maternal MT Treatment Reshapes Gut Microbiota in the HF-Fed Dams and Offspring

**Figure 5:**
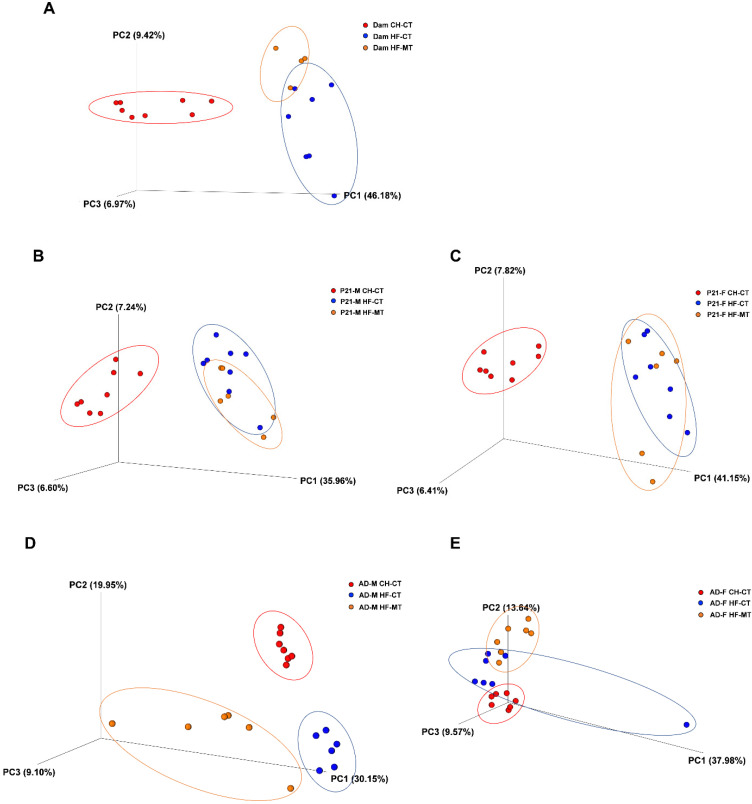
**Maternal metformin treatment reshapes the gut microbiota composition in dams and offspring:** (**A**) principal coordinates analysis (PCoA) 3D plot of unweighted UniFrac distances in 16S rRNA sequencing of fecal contents in dams at weaning; (**B**,**C**) PCoA 3D plots of 16S rRNA sequencing of colonic contents in male and female offspring at weaning; (**D**,**E**) PCoA 3D plots of 16S rRNA sequencing of colonic contents in male and female offspring at 16 weeks old. Each dot represents data from one rat; CH-CT, *n* = 7–8; HF-CT, *n* = 6–8; HF-MT, *n* = 4–7.

Since maternal MT treatment improves the metabolic phenotype and the integrity and inflammatory conditions of the gut in HF-fed dams and offspring, we are curious if the gut microbiota plays a role in it. We investigated the gut microbiota composition using 16S rDNA sequencing assay of fecal bacteria. The sequencing results were analyzed for their beta diversity (reflect species similarity of different groups), as presented in 3D PCoA charts (Figure 5). As expected, significant separation was observed in the CH-CT and HF-CT groups in dams at weaning (ANOSIM, *p* = 0.002, *R* = 0.516) (Figure 5A, Table 2), and this separation was also observed in both male (Figure 5B,D) and female (Figure 5C,E) offspring at weaning and in adulthood (Table 2). The dots representing the HF-MT group were not significantly separated from the HF-CT group in both male (ANOSIM, *p* = 0.412, *R* = 0.001) and female (ANOSIM, *p* = 0.05, *R* = 0.204) pups on PND21 (Figure 5B,C, Table 2). However, the HF-MT group was significantly separated from the HF-CT group in dams (ANOSIM, *p* = 0.044, *R* = 0.286) and adult male (ANOSIM, *p* = 0.006, *R* = 0.32) and female (ANOSIM, *p* = 0.003, *R* = 0.465) offspring (Figure 5A,D,E, Table 2). 

The parameters for alpha diversity analysis are presented in Figure S1 (Chao1 for community richness, Shannon index and Simpson index for community diversity). In both dams and weaning pups, maternal HF diet and MT treatment significantly decreased Chao1 index, and this effect lasted into adulthood (Figure S1A). In P21 pups, the Shannon index was significantly decreased by maternal HF diet and MT treatment (Figure S1Bii,iiii). However, it did not differ among groups in dams and adult offspring (Figure S1Bi,iii,iiiii). No differences in the Simpson index were presented among dams and offspring in their experimental groups (Figure S1C).

To identify species that characterize each experimental group, CH-CT versus HF-CT, or HF-CT versus HF-MT group, we performed LEfSe analysis in dams (Figure S2) and adult offspring (Figure S3). Surprisingly, we found that the control adult male offspring were characterized by a number of species belonging to genera Lactobacillus (Figure S3A), which also showed relative high abundance in the HF-MT group compared with the HF-CT group (Figure S3C). However, in adult female offspring, the CH-CT group and the HF-MT group were both characterized by the genera Clostridium compared with the HF-CT group (Figure S3B,D). Interestingly, we found the abundance of Clostridium was also significantly greater in the control dams compared to the HF-fed dams (Figure S2). The data of beta diversity and LEfSe suggest that maternal MT treatment has long-term effect on offspring’s gut microbiota composition and increases the abundance of some beneficial microbes in adult offspring. 

### 3.4. Maternal MT Treatment Improves Gene Expression of Hypothalamic Inflammatory and Appetite Markers in Offspring from HF-Fed Dams and Restores Hypothalamic Gene Expression of Bile Acid Receptor-TGR5 in Adult Male Offspring

**Figure 6:**
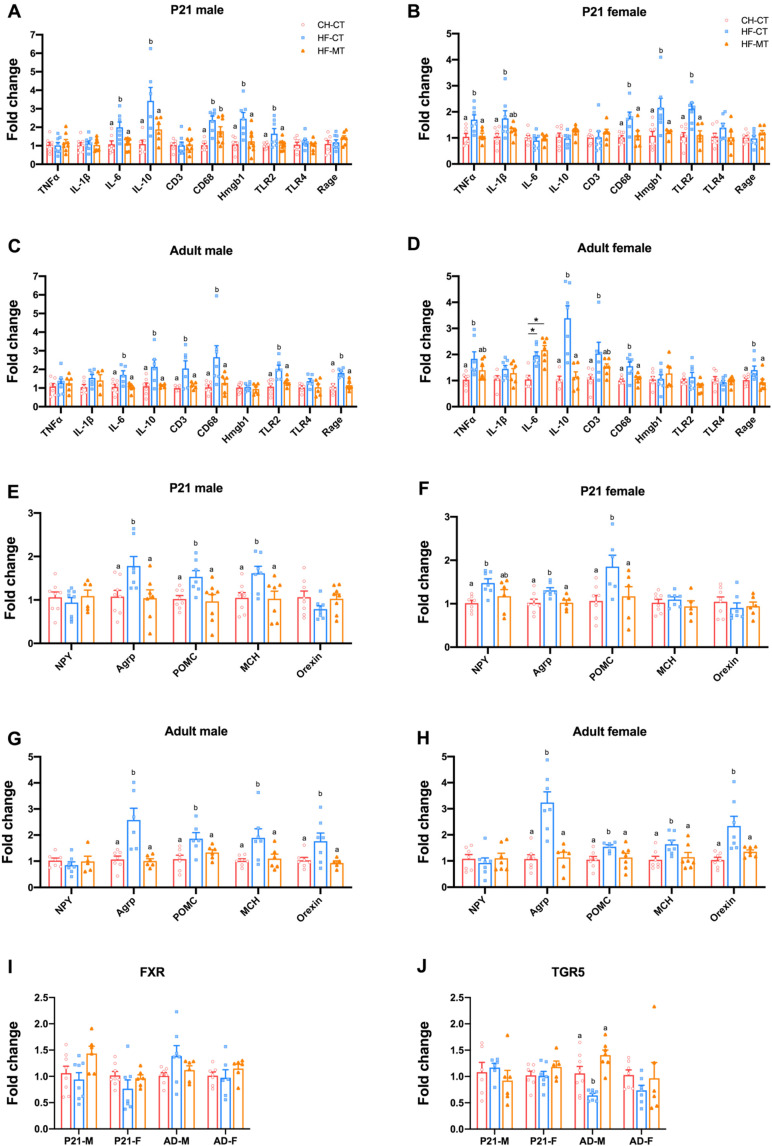
**Maternal metformin treatment improves hypothalamic gene expression of inflammatory and appetite markers in offspring from high-fat fed dams and restores hypothalamic gene expression of bile acid receptor-TGR5 in adult male offspring:** (**A**,**B**) hypothalamic mRNA expression of inflammatory markers in male and female pups at weaning; (**C**,**D**) hypothalamic mRNA expression of inflammatory markers in male and female offspring at 16 weeks old; (**E**,**F**) hypothalamic mRNA expression of appetite markers in male and female pups at weaning; (**G**,**H**) hypothalamic mRNA expression of appetite markers in male and female offspring at 16 weeks old; (**I**) hypothalamic mRNA expression of farnesoid X receptor (FXR) of male and female offspring at postnatal day (P) 21 and adulthood (AD); (**J**) hypothalamic mRNA expression of the G protein-coupled membrane receptor 5 (TGR5) of male and female offspring at P21 and AD. Data are presented as the mean ± SEM. CH-CT, *n* = 5–8; HF-CT, *n* = 6–8; HF-MT, *n* = 5–7. Statistical significance was determined using one-way ANOVA with Tukey’s post hoc tests. Groups with different superscript letters differ from each other at *p* < 0.05 level.

In offspring from HF-fed dams of both sexes, hypothalamic gene expression of inflammatory markers was upregulated at both PND21 and 16 weeks old, while maternal MT treatment restored these gene expression levels (Figure 6A–D). 

The hypothalamus plays vital roles in the central regulation of food intake and energy balance. The orexingenic peptides secreted from hypothalamus include neuropeptide Y (NPY, encoded by *Npy*), agouti-related peptide (AgRP, encoded by *Agrp*), melanin-concentrating hormone (MCH, encoded by *Mch*) and orexin (encoded by *Hcrt*), while pro-opiomelanocortin (POMC, encoded by *Pomc*) in the hypothalamus can derive anorexingenic peptides. The hypothalamic gene expression of both anorexingenic and orexingenic peptides was increased in the HF-CT group in male and female offspring at PND21 and 16 weeks of age, while maternal MT treatment normalized mRNA expression levels of these genes (Figure 6E–H). These data suggest that the beneficial effects of maternal MT treatment on hypothalamic inflammatory status and appetite regulation in the offspring persist into adulthood.

Since TGR5 and FXR are expressed in the HYP and have a role in energy homeostasis, and they are both receptors for gut microbiota metabolites-BAs, we next determined the gene expression of TGR5 (encoded by Gpbar1) and FXR (encoded by Nr1h4) in the hypothalamus. At weaning, hypothalamic gene expression of TGR5 and FXR were not affected by maternal HF diet or MT treatment in male and female pups (Figure 6I,J). However, compared with adult male offspring in the CH-CT group, maternal HF diet significantly decreased mRNA expression of TGR5, which was restored by maternal MT treatment (Figure 6J). The TGR5 gene expression was comparable among groups in adult female offspring (Figure 6J). The hypothalamic FXR mRNA expression was not altered by maternal HF diet or MT treatment in adult male and female offspring (Figure 6I). 

### 3.5. Maternal MT Treatment Improves Maternal HF Diet-Induced Microbial BA Dysmetabolism in Adult Male Offspring

**Figure 7:**
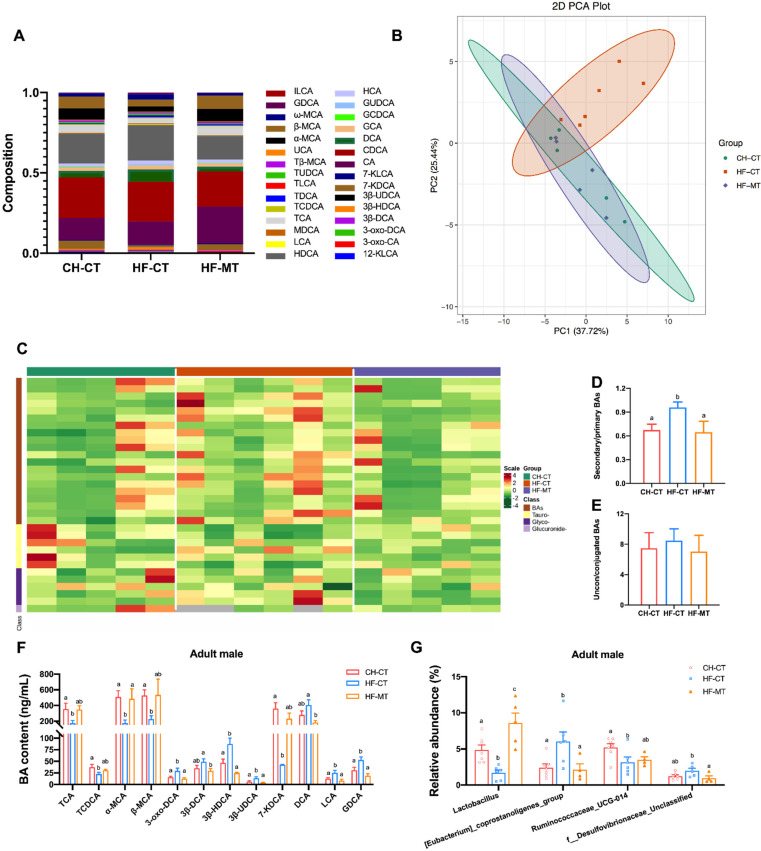
**Maternal metformin treatment improves maternal high-fat diet-induced microbial bile acid dysmetabolism in adult male offspring:** (**A**) BA pool composition in plasma; (**B**) principal component analysis (PCA) plot showing the plasma bile acid (BA) profiles of three experimental groups; (**C**) hierarchical cluster analysis (HCA) plot discriminating the plasma BA profiles of different groups; (**D**,**E**) ratios of secondary/primary and unconjugated/conjugated BAs in plasma; (**F**) significantly altered plasma BAs of three experimental groups; (**G**) relative abundance of the significantly altered gut bacteria at genus levels in fecal contents from the three groups. Data are presented as the mean ± SEM (**D**–**G**). CH-CT, *n* = 5–7; HF-CT, *n* = 5–6; HF-MT, *n* = 4–5. Statistical significance was determined using one-way ANOVA with Tukey’s post hoc tests. Groups with different superscript letters differ from each other at *p* < 0.05 level. (Abbreviations for the subtypes of BAs: I-iso; G-glyco; T-tauro; M-murine; H-hyo; K-keto; U-urso).

Given that BAs play a vital role in regulating gut homeostasis and metabolism, and the hypothalamic gene expression of TGR5 was restored by maternal MT treatment in adult male offspring from HF-fed dams, we analyzed whether maternal MT treatment affects plasma BA levels in adult male offspring. In this study, a total of 30 BAs in plasma were identified by LC-MS across all the experimental groups (Figure 7A). Distinct clustering of the BAs in the CH-CT, HF-CT and HF-MT group was showed by PCA and HCA (Figure 7B,C). The BA metabolite cluster of the HF-MT group almost overlapped with that of the control, while the HF-CT group was significantly separated from the CH-CT group (Figure 7B). Furthermore, the ratio of secondary to primary BAs in the HF-CT group was significantly higher than the control group and was normalized in the HF-MT group (Figure 7D). The ratio of conjugated to unconjugated BAs did not differ among groups (Figure 7E). In addition, maternal HF diet led to reduced levels of tauro-cholic acid (TCA), tauro-chenodeoxycholic acid (TCDCA), α-muricholic acid (α-MCA), β-MCA and 7-keto-deoxycholic acid (7-KDCA) and an increase in the levels of 3-oxo-deoxycholic acid (3-oxo-DCA), 3β-hyodeoxycholic acid (3β-HDCA), 3β-ursodeoxycholic acid (3β-UDCA), lithocholic acid (LCA) and glyco-deoxycholic acid (GDCA) compared with that in the CH-CT group (Figure 7F). However, maternal MT treatment restored these changes to normal levels and decreased the levels of 3β-DCA and DCA compared with the HF-CT group (Figure 7F).

Next, we analyzed the relative abundance of fecal microbiota at the genus level in adult male offspring. As shown in Figure 7G, the relative abundance of *Lactobacillus* and *Ruminococcaceae_UCG-014* was significantly decreased, and that of *[Eubacterium]_coprostanoligenes_group* was increased in the HF-CT group compared with the controls, which were restored by maternal MT treatment. Maternal MT treatment significantly increased the relative abundance of *Lactobacillus* compared with the CH-CT group and decreased the relative abundance of *f_Desulfovibrionaceae_Unclassified* compared with the HF-CT group (Figure 7G). 

## 4. Discussion

Managing maternal overnutrition is an important issue, as it poses an adverse effect on both mother and offspring in short-term and long-term. However, the use of MT treatment for non-diabetic obese pregnant women is not recommended by the American Congress of Obstetricians and Gynecologists because of limited evidence of benefits [35]. In some studies, childhood obesity is commonly observed in models of maternal MT treatment in GDM and PCOS pregnancies in human [11,14,15,16] and in obese pregnancy in rodents [19]. Contrarily, the beneficial effects of maternal MT exposure were observed in maternal haemodynamics in GDM pregnancies [36] and in maternal metabolic health and cognitive function in GDM mouse model [37], in placental expression of oxidative stress markers in human [38], in fetal liver apoptosis in rats [39] and in glucose tolerance and adipose weight in mice offspring [40]. In our study, we found the body weight of HF-MT dams was significantly lighter than the control dams during gestation and lactation. The litter size (CH-CT: 10.6 ± 0.6 vs. HF-MT: 9.8 ± 1.0) and dams’ food intake were similar between these two groups, while the HF-MT offspring was significantly lighter than the CH-CT at birth. We speculate that the decreased body weight of dams in the HF-MT group during gestation was due to slowed fetal growth. Increased prevalence of small for gestational age was also observed in metformin-treated women with restricted gestational weight gain [41]. During lactation, the dams’ body weight in the HF-MT group was still lower than that in the CH-CT group. As we calculated dams’ adipose weight as a percentage of whole body weight, we infer that the lean mass percentage of dams in CH-CT and HF-MT groups was comparable at weaning. Dams’ food intake was not affected by maternal metformin treatment, and we did not measure how much milk the pups consume during lactation. The pups’ body weight in the HF-MT group at PND7, PND14 and PND21 were all similar to the control group, suggesting that the pups may consume more milk to compensate for the low birth weight. Thus, we propose that the decreased body weight in HF-MT dams during lactation may be caused by increased energy expenditure. 

In this study, maternal HF diet showed a significant deleterious effect on offspring’s metabolic phenotype and could cause colonic inflammation and gut-barrier disruption by reshaping the gut microbiota and microbe related BA metabolism. The daily oral administration of MT to HF-fed dams during gestation and lactation reversed the dysbiosis of gut microbiota in both dams and offspring and restored plasma BA composition in adult male offspring, which could further activate TGR5 in the hypothalamus. The hypothalamic TGR5 expression in adult male offspring was restored by maternal MT treatment, which could regulate hypothalamic appetite-related peptides expression and alleviate inflammation, thereby improving offspring’s metabolic phenotype. 

Studies have shown that the gut microbiota is closely associated with the development of obesity and behavioural disorders [42]. Alterations of gut microbiota composition in early life can substantially modify the host’s metabolism, adiposity, energy homoeostasis and the central appetite mechanism, which might cause metabolic disorder and diabetes [43]. The gut microbiota of offspring is highly dysregulated by maternal HF diet in different animal models [44,45,46]. *Lactobacillus*, *Firmicutes*, *Verrucomicrobia*, *Ruminococcaceae*, *Akkermansia* and *Eubacterium_coprostanoligenes_group* are the most commonly HF diet-affected genera [45,47,48,49], which are consistent with our results in adult male offspring. Studies have shown that modulation of gut microbiota by dietary interventions, such as probiotics, prebiotics or fecal microbiota transplantation can restore metabolic imbalances in metabolic disorders such as obesity [50,51,52]. In this study, we show that maternal MT treatment present long-term effect in reshaping the gut microbiota of adult offspring, improving the integrity and inflammatory status of the intestine and decreasing body weight and adipose depots in offspring from HF-fed dams. Previous researches demonstrate the beneficial effects of prenatal MT exposure on offspring’s metabolic phenotype in a maternal or postweaning HF diet model [17,40,53]. However, few studies report the effect of maternal MT treatment on maternal dysbiosis [39]. Our results of beta diversity in the gut microbiota of both dams and offspring suggest that, maternal MT treatment can separate the gut microbiota composition in HF-fed dams from an unhealthy state, and this effect can be transmitted vertically to their offspring and continue to adulthood. In a genetic mouse model of obesity, prenatal MT exposure similarly modified gut microbiota composition in adult offspring of both sexes [18]. However, the LEfSe analysis of this study shows that species characterize maternal MT treatment versus maternal HF diet were sex-specific in adult offspring. Different animal models and postweaning diets in these two studies may lead to the differences in gut microbiota composition in adult offspring. 

BAs are gut metabolites and function as endogenous signaling molecules that regulate innate immune function and host metabolic processes through specific receptors, including FXR and TGR5 [54]. The gut microbiota transforms BA through numerous reactions, such as hydrolysis of conjugated BA to unconjugated free forms by bile salt hydrolases (BSH), forming DCA and LCA through gut microbial 7-dehydroxylation and forming iso- or oxo-BAs through epimerization and oxidation of hydroxyl groups at the target positions [54]. BSH is active in *Lactobacillus* and controls bacterial fitness and host colonization [55]. Free BA produced by BSH can reduce the reabsorb efficiency of intestinal lipids, and BSH over-expressing *E.coli* reduce host weight gain and plasma cholesterol by reducing intestinal cholesterol absorption [56]. In this study, the abundance of *Lactobacillus* is reduced by maternal HF diet in adult male offspring, suggesting decreased level of BSH in the gut, which is associated with reduced gut-barrier integrity and increased inflammation [57]. Moreover, *Lactobacillus* was also found to be the characterized species in CH-CT and HF-MT group of adult male offspring, suggesting a positive role *Lactobacillus* plays in the gut. Furthermore, reduced abundance of *Lactobacillus* and *Ruminococcaceae_UCG-014* and enriched *Desulfovibrionaceae* were also observed in HF diet rodent model in other studies [58,59], which support our results. In colon, gut microbiota deconjugates and dehydroxylates the primary BAs (CA and CDCA) into secondary BAs (DCA and LCA), and increased levels of total BAs were induced by HF feeding and highly correlated with the modulation of gut microbiota [60]. Consistent with our results in adult male offspring, increased levels of DCA were found in dietary or genetic adult obesity models [61]. Bacteria in *Eubacterium* genera have been identified with capability to produce secondary BAs [62]. Here we found increased abundance of *Eubacterium_coprostanoligenes_group* in the HF-CT male offspring. Correspondingly, the ratio of secondary to primary BAs in HF-CT offspring was increased as well. Interestingly, a high-ratio of secondary to primary BAs are linked to decreased risk of obesity in HF-fed mice models [63,64], but we found no direct evidence indicating the specific function of the altered BAs in the HF-CT male offspring. Moreover, in our case, maternal MT treatment restored the composition of the bile acid pool by reshaping the microbiome in adult male offspring, which might contribute to the improved metabolic phenotype.

Several studies report that circulating BAs can reach the CNS and exert dedicated functions by activating receptor FXR or TGR5 [65,66]. Recent studies show that hypothalamic TGR5 signaling is required for the central anorexigenic actions of BAs and exert anti-obesity effects [31,66]. The most potent ligands for TGR5 are LCA and DCA [67], which were elevated by maternal HF diet in adult male offspring in this study. However, the hypothalamic TGR5 expression in adult male offspring was downregulated by maternal HF diet, suggesting that the hypothalamic BA-TGR5 signaling is blunted. Besides, TGR5 activation also attenuates neuroinflammation in several studies [68,69]. Consistently, in this study, maternal MT treatment restored the hypothalamic TGR5 expression and attenuated the inflammatory status in the HYP. Moreover, hypothalamic expression of orexigenic peptides including AgRP, MCH and orexin was increased by maternal HF diet, but reversed back to normal by maternal MT treatment. As reported in previous studies, the activation of TGR5 decreases orexigenic AgRP/NPY release in the HYP [31], we suppose that the blunted TGR5 signaling may contribute to the altered expression of appetite markers in the HYP of adult male offspring, which then affects the metabolic phenotype. In the current study, we failed to measure the accurate food intake in the offspring after weaning, so it is unknown whether maternal HF diet increases offspring’s food intake by suppressing TGR5 signaling in the HYP. However, it is shown that maternal HF-high sugar diet can result in hyperphagia on a HF diet or increased rebound feeding of a basal chow diet following a fast in mice [70]. Recent studies show that maternal gut microbiome influences fetal neurodevelopment during critical prenatal periods through signaling by microbially modulated metabolites to neurons in the developing brain [26]. The hypothalamic feeding circuits are established during the second week of lactation [33]. Thus, the long-lasting beneficial effect of maternal MT treatment on male offspring may be mediated through the gut microbiota-BAs-TGR5 axis during the critical periods of hypothalamic neurodevelopment. 

However, the altered hypothalamic gene expression of TGR5 was only found in adult male offspring from HF-fed dams. In females, HYP TGR5 or FXR expression was not altered by maternal HF diet or MT treatment, suggesting BA signaling may not play important roles in female offspring. However, the genera *Clostridium* that characterize HF-MT adult female offspring was found closely related to MT treatment in mice [71] and human [72] and correlated with branched-chain amino acid metabolism [72]. As reported by previous studies, gut microbiota metabolites other than BAs may play a vital role in regulating host metabolism in female rats [73].

## 5. Conclusions

In summary, we found that maternal MT treatment could improve the integrity of gut-barrier, colonic inflammation and gut dysbiosis induced by maternal HF diet in both dam and offspring by remodeling of the gut microbiota. Furthermore, our findings provide a novel therapeutic mechanism of maternal MT treatment, which can prevent the development of metabolic syndrome in male offspring from HF-fed dams. We also reveal an important role of gut microbiota-BAs-HYP axis in the regulation of male offspring’s metabolic phenotype. In addition, maternal MT treatment modulates offspring’s gut microbiota in a sex-specific manner. 

## Figures and Tables

**Table 1:** Primers used in the quantitative real-time PCR.

Gene	Forward Primer	Reverse Primer	Gene Bank No.
*Actb*	CTATCGGCAATGAGCGGTTCC	TGTGTTGGCATAGAGGTCTTTACG	NM_031144.2
*Tjp1*	AGCGAAGCCACCTGAAGATA	GATGGCCAGCAGGAATATGT	NM_001106266.1
*Ocln*	CTGTCTATGCTCGTCATCG	CATTCCCGATCTAATGACGC	NM_031329.3
*Cldn4*	CGAGCCCTGATGGTCATCAG	CGGAGTACTTGGCGGAGTAG	NM_001012022.1
*Muc2*	ACCATGGGGCTGCCACTA	GATCTTCTGCATGTTCCC	XM_03910270.1
*Tnfa*	GTCGTAGCAAACCACCAAGC	TGTGGGTGAGGAGCACATAG	NM_012675.3
*Il1b*	GCAATGGTCGGGACATAGTT	AGACCTGACTTGGCAGAGA	NM_031512.2
*Il6*	TCCGCAAGAGACTTCCAGCCAGT	AGCCTCCGACTTGTGAAGTGG	NM_012589.2
*Il10*	TGCGACGCTGTCATCGATTT	GTAGATGCCGGGTGGTTCAA	NM_012854.2
*Cd3*	GTCCGGTGACTTGCCTCTAC	CTAGATGCCTGATGCTGGTGT	NM_007648.5
*Cd68*	ACTGGGGCTCTTGGAAACTACAC	CCTTGGTTTTGTTCGGGTTCA	NM_001031638.1
*Hmgb1*	CTAGCCCTGTCCTGGTGGTATT	CCAATTTACAACCCCCAGACTGT	NM_012963.3
*Tlr2*	GCACTTGAGCGAGTCTGCTTTC	GAACAAATAGAACTGGGGGATGTG	NM_198769.2
*Tlr4*	GGCTGTGGAGACAAAAATGACCTC	AGGCTTGGGCTTGAATGGAGTC	NM_019178.2
*Ager*	ACAGAAACCGGTGATGAAGGA	TGTCGTTTTCGCCACAGGAT	NM_05333.6
*Npy*	CTATCCCTGCTCGTGTGTTTGG	TGGTGATGAGATTGATGTAGTGTCG	NM_012614.1
*Agrp*	TGAAGAAGACAGCAGCAGAC	TTGAAGAAGCGGCAGTAGC	NM_03650.1
*Pomc*	TGCTTCAGACCTCCATAGAC	GCTGTTCATCTCCGTTGC	NM_139326.2
*Mch*	GAATGGAGTTCAGAATACTGAGTCA	AGCATACACCTGAGCATGTCAAAT	NM_012625.2
*Hcrt*	CCTGCCGTCTCTACGAACTG	GTTACCGTTGGCCTGAAGGA	NM_013179.3
*Nr1h4*	CTGATTGGGCCCTCCCATTT	CAGATTCTGCCCCAGAGGAC	NM_021745.1
*Gpbar1*	TACTCACAGGGTTGGCACTG	CAAAAGTTGGGGGCCAAGTG	NM_177936.1

*Actb*, beta actin; Tjp1, tight junction protein 1; *Ocln*, occlucin; *Cldn4*, claudin 4; *Muc2*, mucin 2; *Tnfa*, tumor necrosis factor alpha; *Il1b*, interleukin 1 beta; *Il6*, interleukin 6; *Il10*, interleukin 10; *Hmgb1*, high mobility group protein 1; *Tlr2*, toll-like receptor 2; *Tlr4*, toll-like receptor 4; *Ager*, advanced glycosylation end product-specific receptor; *Npy*, neuropeptide Y; *Agrp*, agouti-related peptide; *Pomc*, pro-opiomelanocortin; *Mch*, melanin-concentrating hormone; *Hcrt*, orexin-A; *Nr1h4*, farnesoid X receptor (FXR); *Gpbar1*, G protein-coupled bile acid receptor 1 (TGR5).

**Table 2:** ANOSIM analysis.

	Group1	Group2	*R*-Value	*p*-Value
Dam	CH-CT	HF-CT	0.516	0.002
	CH-CT	HF-MT	0.956	0.004
	HF-CT	HF-MT	0.286	0.044
PND21 male	CH-CT	HF-CT	0.939	0.001
	CH-CT	HF-MT	0.988	0.001
	HF-CT	HF-MT	0.001	0.412
Adult male	CH-CT	HF-CT	0.539	0.004
	CH-CT	HF-MT	0.518	0.002
	HF-CT	HF-MT	0.32	0.006
PND21 female	CH-CT	HF-CT	1	0.001
	CH-CT	HF-MT	0.993	0.001
	HF-CT	HF-MT	0.204	0.05
Adult female	CH-CT	HF-CT	0.449	0.002
	CH-CT	HF-MT	0.875	0.002
	HF-CT	HF-MT	0.465	0.003

Similarity of each of the pairing groups is shown with *R* and *p*-values; *R* value close to 1 indicates that the differences between groups in greater than the differences within each group; *p* < 0.05 represents significance.

## Data Availability

The data presented in this study are available on request from the corresponding author.

## Supplementary Materials

The following supporting information can be downloaded at: https://www.mdpi.com/article/10.3390/nu14173612/s1, Figure S1: Changes of alpha diversity parameters after maternal high-fat diet and metformin treatment in dams and offspring; Figure S2: LEfSe (Linear discriminate analysis Effect Size) plots to identify species that characterize each experimental group in dams at weaning; Figure S3: LEfSe (Linear discriminate analysis Effect Size) plots to identify species that characterize control groups and effects of maternal metformin treatment on adult offspring of maternal high-fat (HF) diet.
