# Supplementary material for: Maternal Treatment with Metformin Persistently Ameliorates High-Fat Diet-Induced Metabolic Symptoms and Modulates Gut Microbiota in Rat Offspring
Source: Nutrients. 2022 Sep 1;14(17):3612. doi: 10.3390/nu14173612 (PMC9460832; doi:10.3390/nu14173612)
Supplement: Supplementary file 1 [file nutrients-14-03612-s001.zip › nutrients-1830347-supplementary.pdf]

Supplemental data:

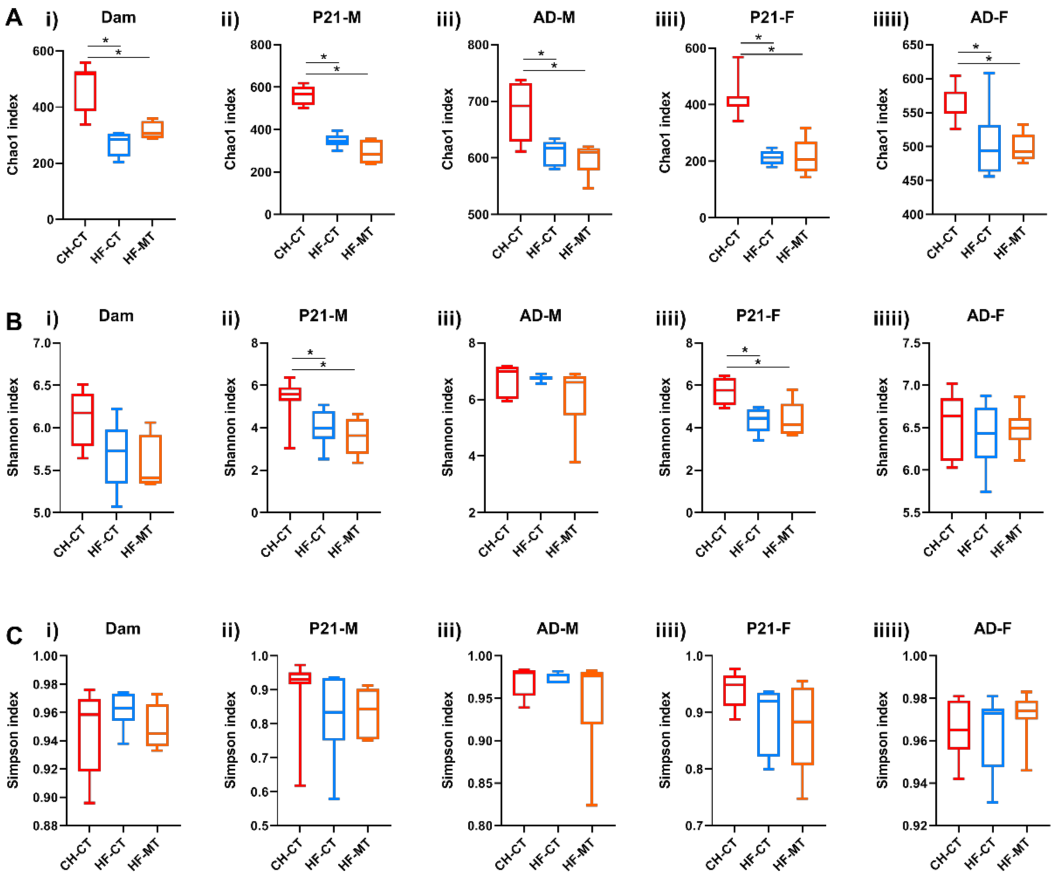

Figure S1. Changes of alpha diversity parameters after maternal high-fat diet and metformin treatment in dams and offspring. (A) Chao1 index for dams at weaning, and male (M) and female (F) offspring at postnatal day (P) 21 and adulthood (AD). (B) Shannon index for dams at weaning, and M&F offspring at P21 and AD. (C) Simpson index for dams at weaning, and M&F offspring at P21 and AD. Data are presented as box plots with medians (middle lines), 25th to 75th percentiles (box boundaries), and minimum and maximum values (whiskers). Statistical significance was determined using one-way ANOVA with Tukey tests for multiple comparisons. $^{*}P < 0.05$. n=6-8.

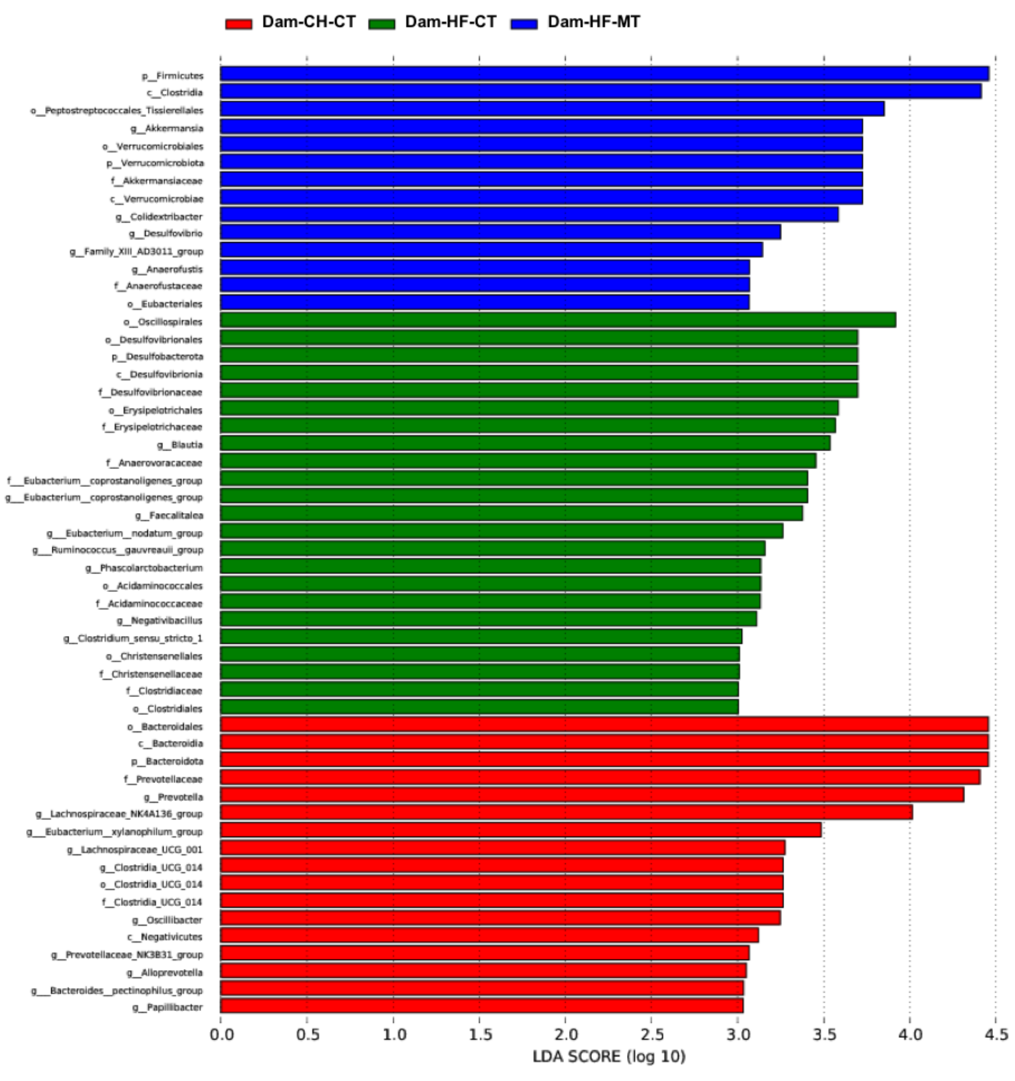

Figure S2. LEfSe (Linear discriminate analysis Effect Size) plots to identify species that characterize each experimental group in dams at weaning. The logarithmic LDA score threshold was set at 3.0. CH-CT, n=8; HF-CT, n=7; HF-MT, n=6.

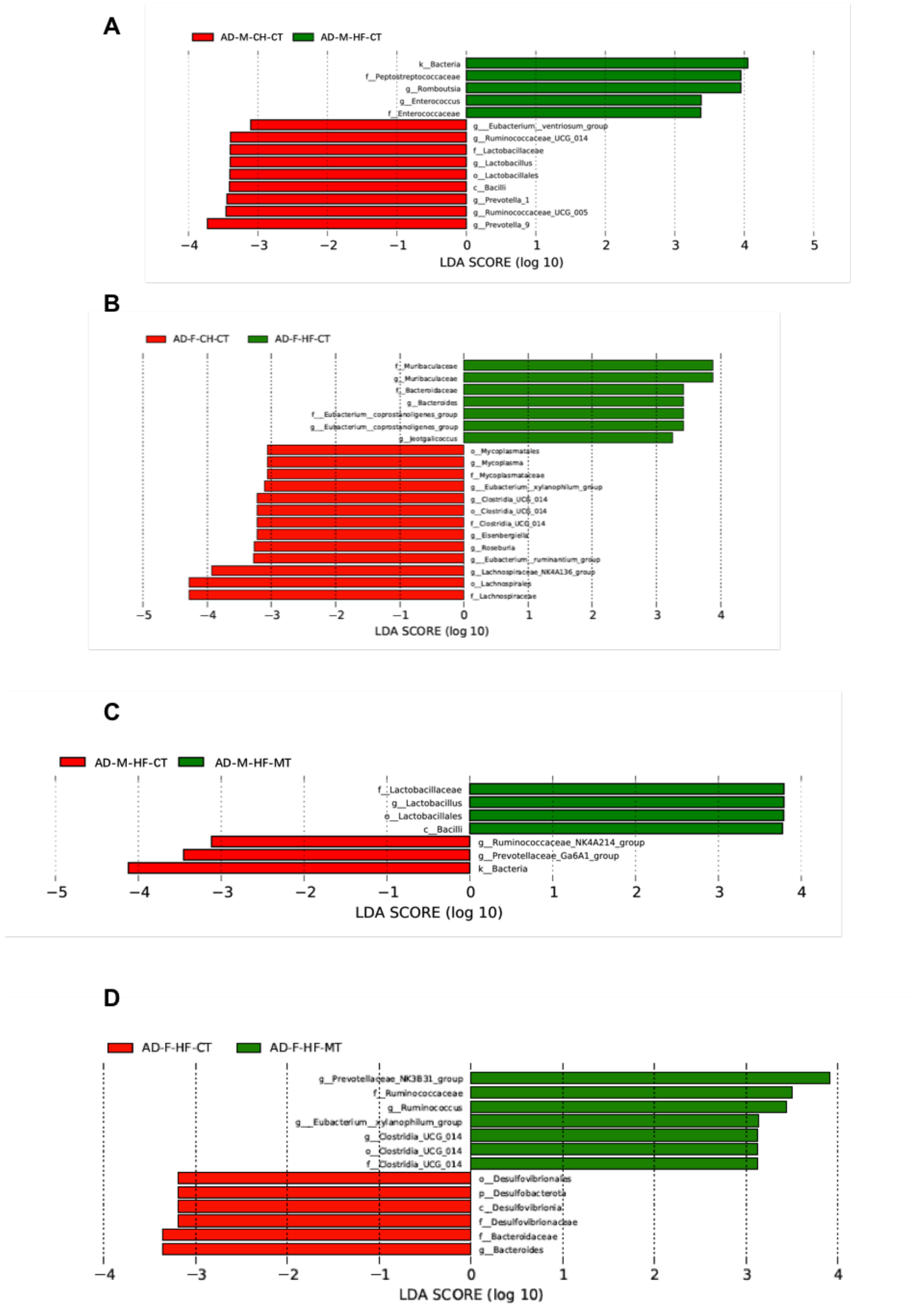

Figure S3. LEfSe (Linear discriminate analysis Effect Size) plots to identify species that characterize control groups and effects of maternal metformin treatment on adult offspring of maternal high-fat (HF) diet. (A, B) LEfSe plots

showing species that characterize control group and effect of maternal HF diet on adult male (A) and female (B) offspring. (C, D) LEfSe plots showing species that characterize effect of maternal metformin treatment on adult male (C) and female (D) offspring of maternal HF diet. The logarithmic LDA score threshold was set at 3.0. n=6-8.
